# M.A.B. Economy: Its Excellent Results

**Published:** 1917-05-12

**Authors:** 


### FOOD IN WARTIME.*

### M.A.B. Economy: Its Excellent Results.

The Metropolitan Asylums Board furnishes an able and highly interesting report of certain alterations which have been made during the last four months in boarding the staff of over 4,000 persons engaged in the institutions under their control. Struck by the grave variations in the cost of feeding staff at different institutions, by the variations in the quantities of the foodstuffs consumed, and by the poor results as regards the quality of the meals in some institutions as compared with others, they determined upon establishing a common menu to be used in all their institutions for four weeks at a time. By the use of these common menus an immense deal of light has been thrown on the methods which give the best results in feeding large numbers. With the first menu formulæ were circulated for the preparation of various dishes, and a table of quantities, which had in practice been found ample for the feeding of 200 persons, was put forward as a guide. After the first month a statement showing the consumption of the different articles of diet per head per day was circulated to all the institutions, with a comment on the remarkable discrepancies it disclosed. This return is now circulated every month, with the effect that the wasteful centres are coming by degrees into line with those managed economically. Unnecessary issues of food have been rigidly put down, greatly to the depletion of the pig-tub; methods of cooking have been improved by advice and instruction, and the value of mechanical appliances brought to the notice of managers. From the very beginning the Board enlisted the co-operation of the officers and matrons of the institutions in drawing up the common menu and initiating reforms. The chairman of the staff dietary section visited and inspected the kitchens when difficulties were reported, and the sympathy and support of all concerned in the experiment were secured.

### Results of the Experiment.

Within the last sixteen weeks the following quantities of foodstuffs have been saved in the dietary of about 4,000 persons as compared with what would have been issued under the old dietary: Milk, 3,726 gallons; eggs, 43,112; tea, 15½ cwt.; sugar, 88 cwt.; flour, 36 cwt.; meat, 411 cwt. The estimated total value of the saving effected during the period is £4,130. But it is interesting to observe that the rate of saving is progressive, rising from the first month, when it was at the rate of £6,900 per annum, to a saving in the fourth month at the rate of £24,500, or (deducting savings fairly attributable to the Food Controller's regulations) of £18,772. When the rise in prices, which continued steadily throughout the whole period, is taken into consideration these figures stand out as a truly amazing testimony to the value of brains in the commissariat.

### The Problem of Rationing.

The Board wisely got into touch with the Food Controller's Department with a view to ascertaining whether the broad average laid down for the public was intended to apply to households consisting solely of adults, and obtained the reply that some modification of the scale might in certain circumstances be permitted. The Board, however, has reached the conclusion that it is possible without undue hardship to bring the consumption at all the institutions more or less to the Controller's level.

The following table shows the average weekly consumption per head at a few institutions (unselected) during the sixteen weeks ending March 24:—

| Bread and Flour. Lbs. per Head per Week | | | | Meat, excluding Fish (except for 1st Period) Lbs. per Head per Week | | | |
|---|---|---|---|---|---|---|---|
| 1st Period | 2nd Period | 3rd Period | 4th Period | 1st Period | 2nd Period | 3rd Period | 4th Period |
| 1. 6.4 | 6.3 | 6.2 | 5.5 | 5.3 | 4.0 | 3.7 | 3.4 |
| 2. 6.7 | 5.8 | 5.5 | 4.8 | 5.1 | 3.6 | 3.4 | 2.9 |
| 3. 6.4 | 6.7 | 6.1 | 4.8 | 5.1 | 3.8 | 3.2 | 2.6 |
| 4. 3.8 | 4.1 | 4.2 | 4.0 | 5 2 | 4.9 | 4.4 | 3.3 |
| 5. 4.1 | 4.3 | 4.1 | 3.6 | 5.2 | 4.4 | 2.8 | 2.6 |
| 6. 4.3 | 4.7 | 5.0 | 5.2 | 5.1 | 4.1 | 3.6 | 3.2 |
| 7. 8.1 | 8.1 | 8.0 | 5.7 | 6.6 | 5.8 | 5.2 | 3.8 |

The Board appears to have succeeded admirably in the aim of improving the dietary, which animated them from the outset no less forcibly than the determination to abolish extravagance. The new methods inaugurated for preparing and serving the food, and the issue of a well-thought-out menu varied according to season, have had the effect of minimising any privations imposed by the national shortage of food, and have levelled up the whole standard of the commissariat. The results of the publicity accorded to these experiments should be far-reaching.

### Mechanical Appliances.

Bad kitchen equipment is responsible for much waste of labour and material in institutions. The Board recommends the use of the "Berkel" machine for cutting up hot joints, slicing bread, etc.; the use of bakers' ovens, which, wherever installed, effect a large saving in gas; and the provision where possible of an electrical mincer and mixer of American manufacture, warranted to make 5 cwt. of pudding (sufficient for 1,000 patients) in fifty minutes. These and other appliances save material, as well as enabling the kitchen staff to be reduced, and would doubtless pay for their own cost within a short time of their installation. But they need a well-instructed person in the kitchen, able to put them to the best use.

---

**Leeds Sanitary Committee has agreed to maintain ten beds at the Leeds Maternity Hospital in consideration of such cases being available when required for complicated cases recommended by the Medical Officer of Health.**

---

* Previous Articles appeared February 24, March 10, 24, and 31, April 14 and 21, May 5.

